# Sedimentation and mobility of PDCs: a reappraisal of ignimbrites’ aspect ratio

**DOI:** 10.1038/s41598-017-04880-6

**Published:** 2017-06-30

**Authors:** Guido Giordano, Domenico M. Doronzo

**Affiliations:** 1 Dipartimento di Scienze, Università di Roma Tre, Roma, Italy; 2 Institute of Earth Sciences “Jaume Almera”, CSIC, Barcelona, Spain; 3 Centro de Geociencias, Universidad Nacional Autonoma de Mexico, Queretaro, Mexico

## Abstract

The aspect ratio of ignimbrites is a commonly used parameter that has been related to the energy of the parent pyroclastic density currents (PDCs). However this parameter, calculated as the ratio between the average thickness and the average lateral extent of ignimbrites, does not capture fundamental differences in pyroclastic flow mobility nor relates to lithofacies variations of the final deposits. We herein introduce the “topological aspect ratio” (ARt) as the ratio of the local deposit thickness (Ht) to the distance between the local site and the maximum runout distance (Lt), where Ht is a proxy for the PDC tendency to deposit, and Lt a proxy for the PDC mobility or its tendency to further transport the pyroclastic material. The positive versus negative spatial gradient d(ARt)/dx along flow paths discriminate zones where PDCs are forced (i.e. where they transport the total energy under the action of mass discharge rate) from zones where they are inertial (i.e. where they transport the total energy under the action of viscous or turbulent fluidization). Though simple to apply, the topological aspect ratio and its spatial gradient are powerful descriptors of the interplay between sedimentation and mobility of PDCs, and of the resulting lithofacies variations.

## Introduction

Low aspect ratio ignimbrites (LARI) are among the most intriguing and less understood deposits on Earth. They were introduced to describe high mobility flows^1^ by rationing the average thickness of the deposit (H) and its horizontal extent (L). Walker^2^ discussed distinctions between high aspect ratio ignimbrites (HARI) and LARI characterized by H/L of 10^−2^–10^−3^ and 10^−4^–10^−5^, respectively. Most large volume ignimbrites (VEI 6 to 8) are LARI, although they encompass a huge spectrum of deposit types in terms of lithofacies associations, relationships with topography, and temperatures of emplacement^1, 3–10^. The mobility of the parent PDCs has been interpreted in terms of extremely high flow velocity and mass discharge rate^1, 11^, within a dominantly turbulent flow dynamics^12^. Current understanding of pyroclastic density currents (PDCs) dynamics describes two end members, concentrated and dilute^13–15^. In order to account for their energy-stratified nature, Doronzo^16^ introduced two new end members of PDCs: forced and inertial. Forced PDCs transport the total energy (combination of density, velocity and thermal energy) under the action of sustained mass discharge rate, on steep slopes and in channelized topography. Inertial PDCs transport the total energy under the action of viscous or turbulent fluidization^17^. These new end members are based on sediment thermo-fluid dynamics, and do not substitute nor overlap with the classic terminology of PDCs. On the other hand, they allow to account for the competition between flow pressure drop (transport) and sedimentation rate, encompassing flow thermal effects that are unique for PDCs among all natural density currents^18, 19^, and the same PDC can transform between forced and inertial in space and time (see Supplementary Material). In this paper, we reapprise the concept of the aspect ratio of ignimbrites, by extending the discussion to specific case studies and testing the forced and inertial end members of PDCs.

## Sedimentation of PDCs

Where and when PDCs are forced, i.e. where and when their internal pressure is supported (see Supplementary Material), they mostly emplace massive-and-chaotic deposits due to increase in particle concentration and poor particle selection in the flow (hindered settling^20^). At the same time, forcing also increases the lateral transport. On the other hand, where and when inertial, i.e. where and when the internal pressure drops, PDCs do not necessarily emplace structured deposits^17, 20, 21^, as inertia depends both on the density and velocity contrasts between the flow and the surrounding atmosphere^17, 18^. The mutual exchange between density and velocity fluxes within the PDC thus affects the final size of transportation (runout) and the onset of deposition (thickness), and variations of the degree of forcing (or its mirroring degree of inertia) of the PDC in space and time make the emplacement of the deposit a combination of progressive aggradation (sequence-scale) and en masse deposition (layer-scale)^13, 16, 17, 21, 22^. Being aware that during a PDC event, fluxes of the total energy wax and wane in space and time^13, 23^, one needs to integrate for the total duration of the PDC in order to use the total extent and thickness variations of the final deposit as proxies for the continuous interplay between transportation and deposition. For this reason it is essential to apply such approach to units for which the internal 3D stratigraphy is well established and deposition at each correlated site can be considered continuous.

## The “topological aspect ratio”

Many ignimbrites share similar low aspect ratios but with very different volumes and field characteristics (Table S1, Supplementary Material), showing that the aspect ratio, as it is defined, does not capture fundamental differences like behavior and mobility of the flow, nor it relates to lithofacies transitions in the deposits. As we want to compare the tendency of the PDC at each site to sediment versus transport, we herein define the “topological aspect ratio” (AR_t_) as the ratio of the local deposit thickness (H_t_) to the distance between that site and the maximum runout distance (L_t_) (Fig. S1, Supplementary Material). L_t_ can be considered for a given deposit as its “potential runout” and varies between the maximum value at the vent and 0 at the maximum runout distance. Usually, the radial distance can be safely assumed as a proxy for the real travel distance, although corrections should be made in specific cases where topographic divergence is substantial. Even the distance of the farthest outcrop (e.g. for ancient ignimbrites) can be used for this purpose as the reference for all localities aligned along the flow path. We therefore consider H_t_ as a proxy for the PDC tendency to deposit, whereas L_t_ as a proxy for the PDC mobility or tendency to further transport the pyroclastic material. The spatial gradient of AR_t_ (dAR_t_/dx) allows to visualize different downcurrent trends (Fig. S1, Supplementary Material). For example, a sheet with constant thickness shows an AR_t_ increasing with distance. Therefore, a positive gradient (i.e. where AR_t_ increases with distance) implies that, while depositing at each site, most of the material is transferred ahead of that site by the surviving PDC. These conditions correspond to a dominantly forced regime, in which both transportation and sedimentation are affected by external drivers (e.g. high mass discharge rate), such as in proximal regions, along steep slopes and in areas of confining topography. On the other hand, a negative gradient (i.e. where AR_t_ decreases with distance) implies that the deposit thins faster than the lateral decrease of the potential runout. This suggests that there is nothing that forces the pyroclastic material from outside the PDC, and internal inertia becomes dominant both for transportation and sedimentation. Intermediate conditions imply a flattening of the AR_t_ gradient and indicate a sort of balance between forced and inertial regimes.

In the following examples, we will use three well-constrained ignimbrite case studies (Pozzolane Rosse, Cerro Galan, Peperino Albano) with a similar regional low aspect ratio, but different in volume, chemistry and degree of paleotopographic control (Table S1, Supplementary Material) to illustrate the value of introducing the topological aspect ratio.

### Application of the topological aspect ratio

**Figure 1:**
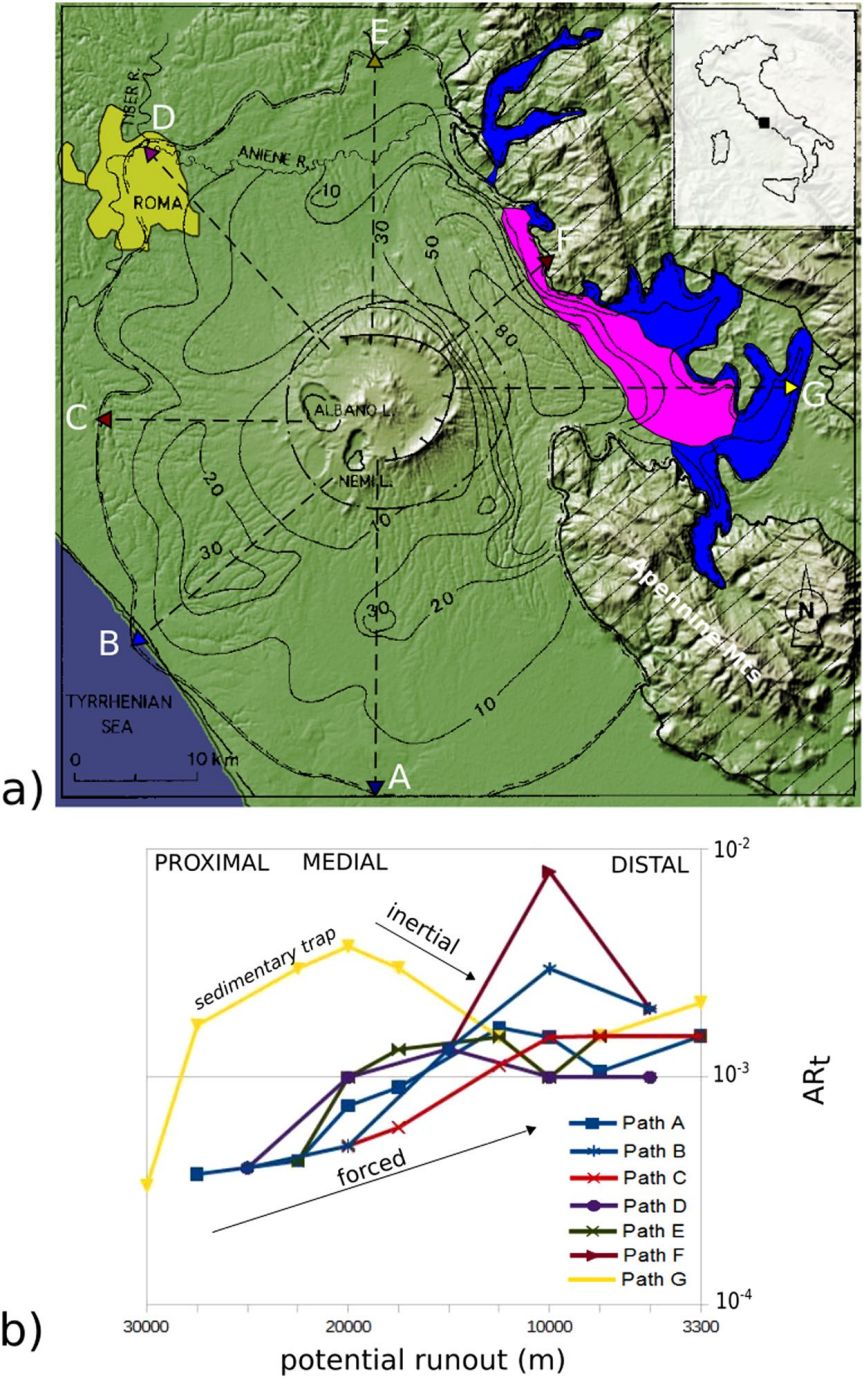
Topological aspect ratio (AR_t_) of the Pozzolane Rosse ignimbrite (Colli Albani, Italy; see Table S1 in Suppl. Material for characteristics); (**a**) Isopach map (in m); arrows indicate paths along which AR_t_ has been calculated (see Fig. S2 and Table S2 in Supplementary Material); pink indicates areas where undulations and cross stratifications occur; blue indicates areas where the ignimbrite is valley-confined; the extent of Roma city is pale green. (Modified from^24^; NASA image created by Jesse Allen, using SRTM data provided courtesy of the University of Maryland’s Global Land Cover Facility, available at http://visibleearth.nasa.gov/view.php?id=37794). – (**b**) Plot of potential runout versus topological aspect ratio AR_t_. The slow rise of AR_t_ with distance indicates forced flow conditions as material is mostly transported; inertial conditons are described by a decreasing AR_t_, e.g. downcurrent respect to sedimentary traps (see text for explanation). (drawing realized with Inkskape ver 0.91 https://inkscape.org).

The Pozzolane Rosse (RED) is a low grade, VEI 7, tephritic ignimbrite (Table S1 in Supplementary Material)^24, 25^. The ignimbrite is radially distributed, with a maximum runout of 33 km, and an average thickness of 21 m that reaches 80 m along the major paleovalley to the E of the volcano (Fig. 1a). The ignimbrite climbed up to 400 m above the surrounding plains. The main lithofacies is massive-and-chaotic (Fig. S3a,b), with the exception of a few localities to the E of the volcano, where stratification can be observed (Figs 1a and S4a,b, Supplementary Material).

In order to obtain the RED topological aspect ratios, we superimposed a squared grid with unit length dimension of 3.3 km to the isopach map (Fig. S2, Supplementary Material). We defined 7 radial profiles to encompass all paleotopographic conditions, i.e. lateral spreading on open and flat topography (N-W-S sectors) and interaction with paleotopographies (E sector; Fig. 1a). The calculated AR_t_ are plotted against distance in Fig. 1b. Values of the topological aspect ratio vary between 10^−4^ and 10^−3^. The gradient of AR_t_ with distance shows an increase from proximal to medial areas common to all paths, which can be related to the transport of the pyroclastic material in a PDC forced by a sustained mass supply rate. This positive trend is mild, concave and extends for 15–20 km in unconfined regions (paths A, B, C, D, E in Fig. 1a). The corresponding deposit thicknesses vary from 10 m in proximal areas to 30 m; lithofacies is everywhere massive-and-chaotic defining a single depositional unit (Fig. S3a,b, Supplementary Material). To the E of the volcano, the positive trend is much steeper, convex and less extended, for about 10 km, as the result of the sudden increase in the deposit thickness (isopach line 80 m in Fig. 1a) along a cross-flow paleovalley (paths F, G in Fig. 1a). From medial to distal areas, the AR_t_ gradients are different for unconfined and topographically-controlled regions. In unconfined regions (paths A, B, C, D, E) AR_t_ gradients become flat. By contrast, paths F and G show a steep negative AR_t_ gradient, just beyond isopach 80 m (Fig. 1b). We interpret this steep negative gradient as the sudden decrease of the forcing of the PDC due to rapid sedimentation inside the paleovalley, which locally subtracted a substantial proportion of the mass transported from the parent flow, decreasing the lateral mass discharge rate and increasing the net effect of inertia. These areas are the only where the otherwise massive-and-chaotic RED ignimbrite shows crude undulated- to cross-stratification (Fig. 1a and Fig. S4a,b, Supplementary Material). Downcurrent RED turns back to massive-and-chaotic as the result of flow channelization in paleovalleys (Fig. 1a). Along profiles A and G, the more distal part of the ignimbrite shows a positive gradient of AR_t_, interpreted as the result of increased sedimentation where the PDC approached its stop at maximum runout distance.

**Figure 2:**
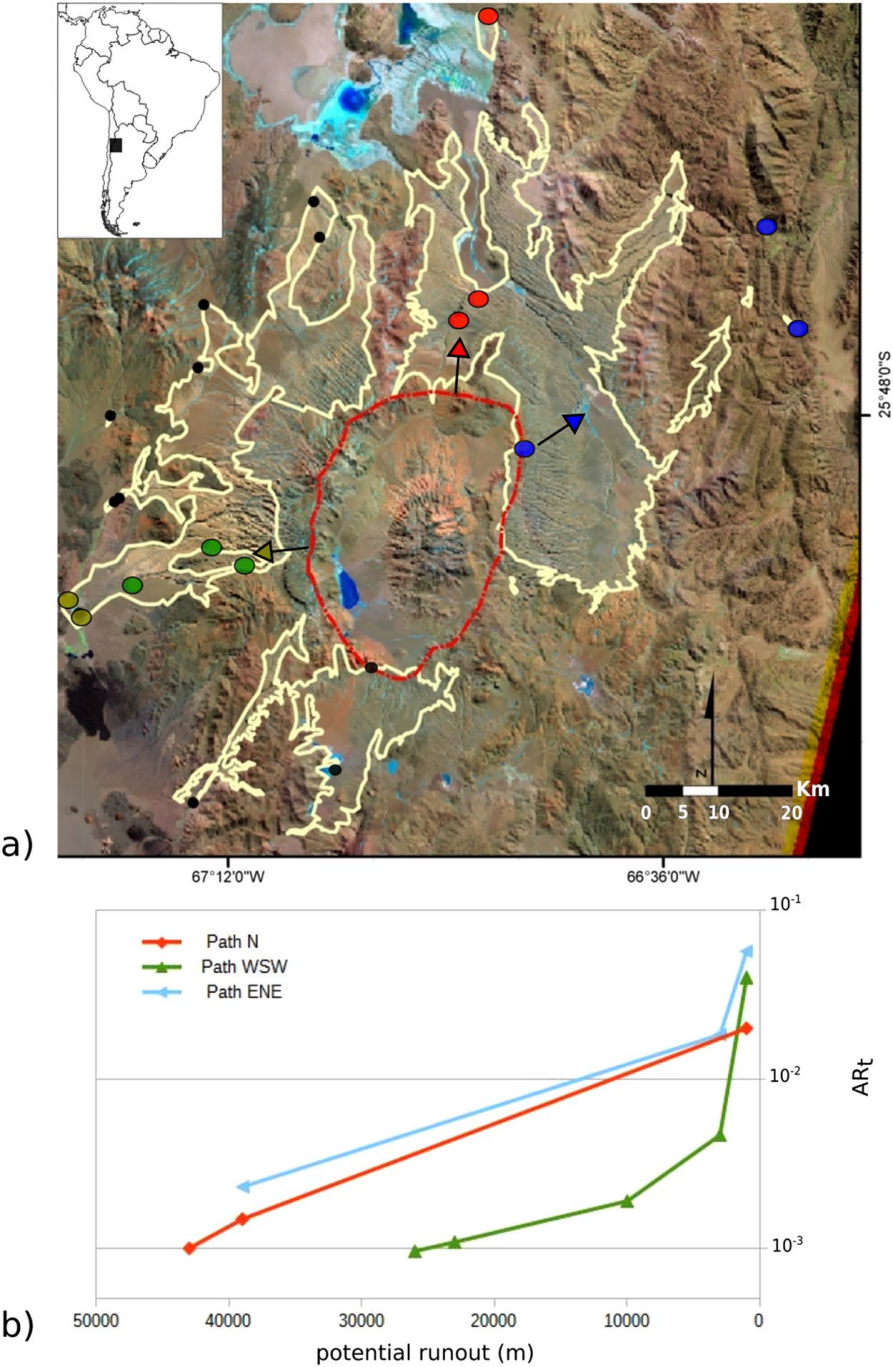
Topological aspect ratio (AR_t_) of the Cerro Galan Ignimbrite (Argentina; see Table S1 in Suppl. Material for characteristics); (**a**) Areal extent of the ignimbrite (yellow line); topographic rim of caldera in dashed red; green, red and blue dots indicate sites with measured sections used for calculation of AR_t_ along paths indicated by arrows (see Table S2 Supplementary Material; data from^9, 26^) – (**b**) Plot of potential runout versus topological aspect ratio AR_t_. The continuous rise of AR_t_ with distance indicates persistent forced flow conditions supported by a high mass flux (see text for explanation). (Landsat imagery courtesy of NASA Landsat Program, 2007, Landsat ETM + scene LE72310792006253COA00, SLC-Off, USGS, Cerro Galan, 10/9/2006; inset map of S America is taken from http://www.d-maps.com/pays.php?num_pay=120&lang=en; drawing realized with Inkskape ver 0.91 https://inkscape.org).

The proposed methodology can be employed where measured stratigraphic sections are available. Another excellent case study is the VEI 8, rhyodacitic Cerro Galan ignimbrite (CGI) (Table S1, Supplementary Material)^9, 26^. The ignimbrite is ubiquitously massive-and-chaotic, and forms a single depositional unit with the exception of few distal localities (Figs S5, S6, S7, Supplementary Material). It is radially distributed, with a maximum radial distance of 74 km (Fig. 2a), and an average thickness of 45 m that reaches a maximum of >100 m. We constructed three AR_t_ profiles for CGI (Fig. 2b). All show a positive AR_t_ gradient which suggests forced flow conditions. Path N maintains its gradient with distance and relates to spreading on an open flat plain. The other two paths become valley confined at their distal ends, where show a steeper positive gradient due to an increase in forcing, because the parent flow was channelized and approached its maximum runout. The distal sites of Path ENE are the only where the CGI shows the aggradation of depositional units with no signs of erosion in between. We suggest that these record an en masse emplacement of the distal lobes as the flow was about to stop. In this case therefore, the absence of evidence for shearing in the deposit indicates that inertia did not play a role in deposition, which is recorded by the steep positive AR_t_ gradient.

**Figure 3:**
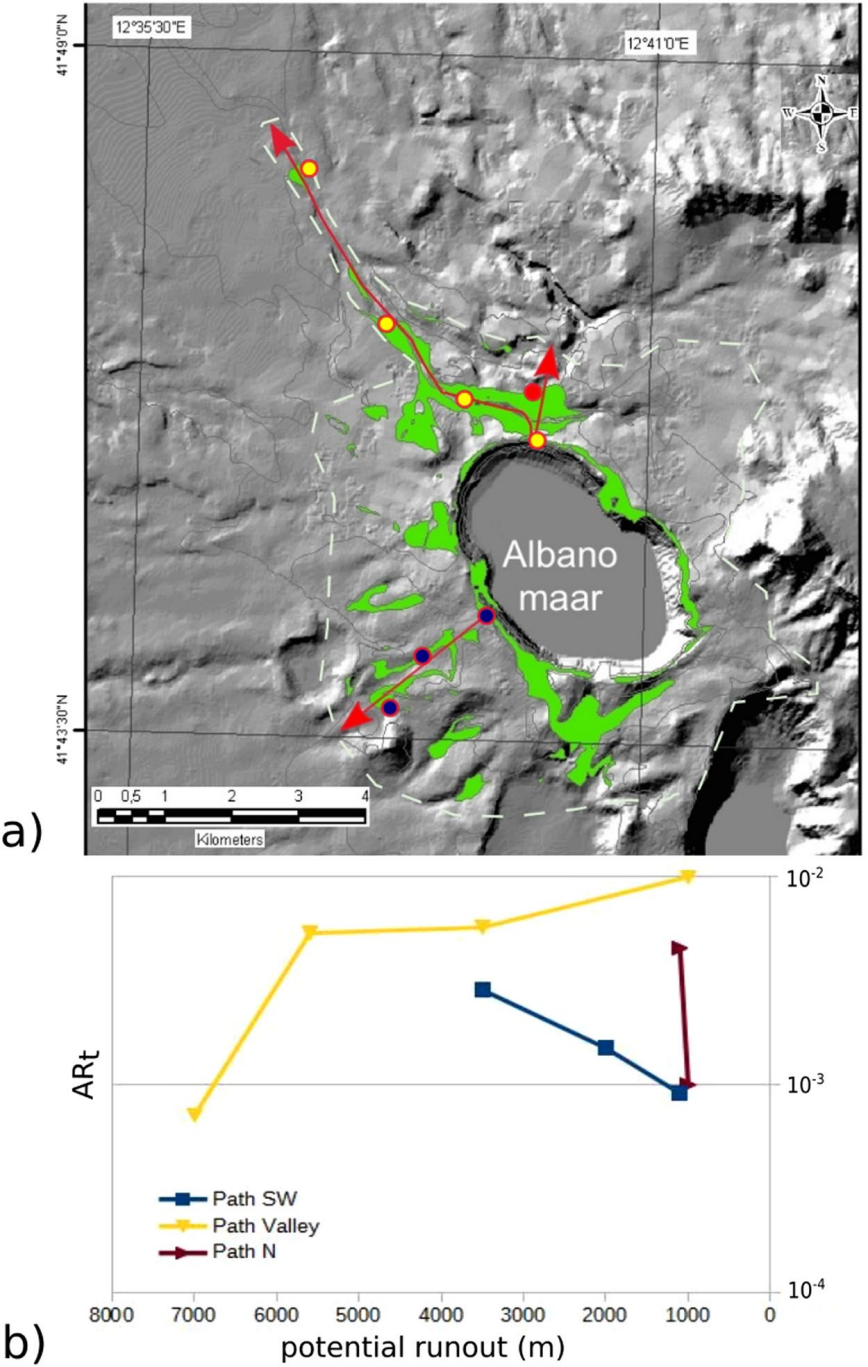
Topological aspect ratio (AR_t_) of the Peperino Albano (Colli Albani, Italy; see Table S1 in Suppl. Material for characteristics; see Fig. 1 for location of Albano maar); (**a**) Areal extent of the phreatomagmatic ignimbrite (outcrops in green; hillshade image elaborated from Digital Terrain Model resolution 30 m available at www.pcn.minambiente.it/GN); dashed white line indicates the envelope of the maximum extent of the deposits; yellow, red and blue dots indicate sites with measured sections used for calculation of AR_t_ along paths indicated by red arrows (see Table S2, Supplementary Material; data from^25, 27^) – (**b**) Plot of potential runout versus topological aspect ratio AR_t_. Path SW and Path N both show a rapid decrease of AR_t_ indicating inertial flow conditions and a rapid dissipation of the internal flow pressure that is by contrast maintained by channellization along the Path Valley, where AR_t_ shows an increase with distance (see text for explanation). (drawing realized with Inkskape ver 0.91 https://inkscape.org).

The third case study is the VEI 4, phreatomagmatic Peperino Albano ignimbrite (PNO; Fig. 3a,b; Table S1, Supplementary Material), which shows both veneer and valley pond lithofacies^27–29^. The valley pond facies developed as a the result of the detachment of an underflow drained in a valley at high angle respect to the PDC radial direction away from the maar. We constructed three AR_t_ profiles for PNO (Fig. 3b). Rapid thinning and negative AR_t_ gradients are observed along the unconfined Path SW and Path N which suggest inertial flow conditions. The negative AR_t_ gradient for Path N is very steep and relates to the drainage of most of the pyroclastic material inside the cross-sectional paleovalley. Accordingly, the main lithofacies along Path SW and Path N is cross stratified (Fig. S8a,b, Supplementary Material). By contrast, Path Valley along the thick valley pond lithofacies shows a steep convex positive AR_t_ gradient, in agreement with forced flow conditions and massive-and-chaotic deposits (Fig. 3b and Fig. S9 Supplementary Material).

## Discussion and Conclusions

**Figure 4:**
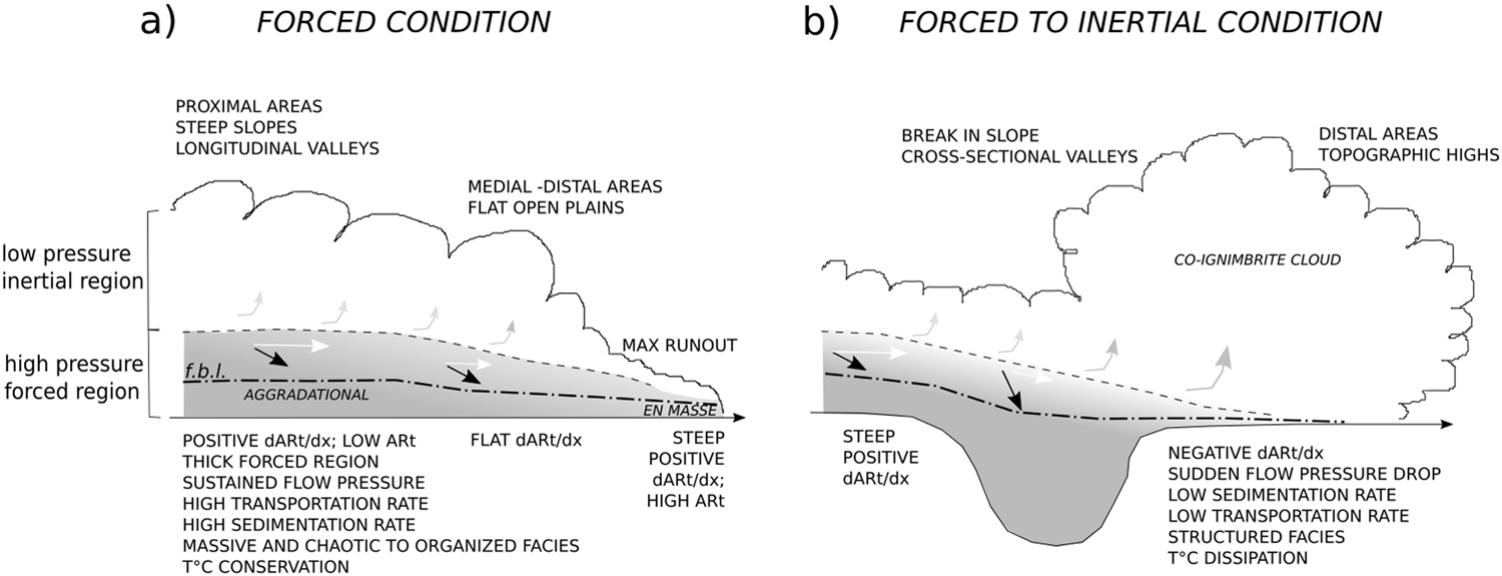
Model explaining AR_t_ gradients of ignimbrites in terms of degree of forcing or inertia of the parent PDCs (see text for explanation). White arrow = sediment transport; black arrow = sediment deposition; their dimensions indicate the relative magnitudes. The ignimbrite lithofacies results from the control of the sediment flux over the position of the flow boundary layer (f.b.l.) respect to the top of the forced region.

The three presented case studies are all examples of LARI, however they clearly show how the commonly used (regional) aspect ratio cannot describe their differences. By contrast, the proposed topological aspect ratio AR_t_ and its gradient well capture major processes related to sedimentation and transportation in forced versus inertial (parts of) PDCs. Such amounts can be calculated both for modern and ancient ignimbrites, providing a powerful use of field measurements like lithofacies variations and thicknesses. Figure 4 summarizes the main PDC processes that can be inferred from the spatial gradient of the topological aspect ratio (dAR_t_/dx): (i) Mild positive AR_t_ gradients and low AR_t_ values describe forced PDCs, related to sustained flow pressure at high mass discharge rates (e.g. in caldera-forming eruptions; in forcing topography, such as steep downslopes or longitudinal valleys); the forced region of the PDC includes both the transportational and depositional systems, so that the flow boundary layer (f.b.l.) is well inside this region (Fig. 4a) [ref. 13]; deposits are aggraded to tabular geometries (e.g. constant thickness or increasing with distance), and are dominantly massive-and-chaotic, although internal organization and cross-stratification are possible where velocity flux is high; (ii) Flat AR_t_ gradients describe zones where forcing and inertia are balanced, such as in medial-distal areas where PDCs spread on flat and open topographies, progressively dropping flow pressure and mass via deposition; the forced region thins and the f.b.l. is closer to the inertial region of the flow; deposits have mildly decreasing thicknesses with distance, and can be massive-and-chaotic to internally organized; (iii) Negative AR_t_ gradients describe zones where the PDC becomes dominantly inertial, such as in pyroclastic surges, above topographic highs, or where the flow pressure drops via rapid sedimentation, such as beyond topographic obstacles (Fig. 4b); the inertial region, where turbulence dominates, is therein very close to directly in contact with the f.b.l.; deposits have strong decreasing thicknesses with distance, and are internally organized to structured.

A major outcome of our analysis is that the so called LARI encompass a wide range of flow behaviors^1, 4, 6, 8, 9, 11, 30^ and this explains why previous authors had not found a simple correlation between regional AR and volume^31^. The AR_t_ gradients allow the interpretation of thickness and lithofacies variations with distance and topography, and show that forcing is the major factor affecting the parent flow mobility, where the high mass flux allows to sustain the internal flow pressure (reducing its drop) in space and time though at high sedimentation rates, also maintaining the relative thermal insulation of most known large volume ignimbrites.

## Electronic supplementary material

Supplementary Material
