# Supplementary material for: Sedimentation and mobility of PDCs: a reappraisal of ignimbrites’ aspect ratio
Source: Sci Rep. 2017 Jun 30;7:4444. doi: 10.1038/s41598-017-04880-6 (PMC5493644; doi:10.1038/s41598-017-04880-6)
Supplement: Supplementary file 1 — Supplementary Material [file 41598_2017_4880_MOESM1_ESM.doc]

**Supplementary Material**

**Sedimentation and mobility of PDCs: a reappraisal of ignimbrites' aspect ratio**

Guido Giordano^1^*, Domenico M. Doronzo^2,3^

^1^Dipartimento di Scienze, Università di Roma Tre, Roma, Italy

^2^Centro de Geociencias, Universidad Nacional Autonoma de Mexico, Queretaro, Mexico

^3^Institute of Earth Sciences ‘‘Jaume Almera’’, CSIC, Barcelona, Spain

*corresponding author: email: [guido.giordano@uniroma3.it](mailto:guido.giordano@uniroma3.it)

In order to account for the energy-stratified nature of PDCs, particularly at local topographic sites (ramps, valleys, breaks in slope), we combine density and velocity into a local mass discharge rate of the pyroclastic flow, having dimension of [kg s^-1^]. This is because a mass flux and a velocity flux mutually occur through the flow within the current, affecting the size of sedimentation and transportation. Fluid dynamically, these fluxes define the current’s flow pressure, which can be sustained (or dropped) by the local mass discharge rate. In this way, we link the ‘‘forced regime’’ of PDCs to a slower drop of the flow pressure, while the ‘‘inertial regime’’ to a faster one. For the same PDC event, the two regimes can occur at different topographic sites, or one can transform into the other depending on the local mass discharge rate that sustains the PDC fluidization (e.g. Doronzo, 2012^16^; Roche et al., 2013^17^; Sulpizio et al., 2014^14^). The flow pressure supports transportation, while its drop supports sedimentation, which is why the ‘‘topological aspect ratio’’ in the present paper can effectively relate to the forced and inertial regimes of PDCs. This and the following development are the theoretical and analytical bases where we build the topological aspect ratio on.

According to Doronzo et al. (2016)^19^, we can analytically relate the local mass discharge rate of the pyroclastic material () and the pyroclastic flow temperature (*T_flow_*) through the following

where *t* is the flow time, Ω is the control volume, *ρ_p_* is the particle density, *T_p_* is the particle temperature, and *T_g_* is the gas temperature. At the local site where the pyroclastic deposit is emplaced, the inertial fraction must be zero

while the pyroclastic deposit temperature (*T_dep_*) strictly depends on the pyroclastic material accumulated on the ground, so

This equation states that the deposit temperature depends on the mass flux that effectively contributes to build up the final deposit, meaning that all involved parameters refer to the local deposit and to the particles. Therefore, we can write the control volume as Ω = *h*·*Sup*, where *h* is the deposit thickness (from isopach), and *Sup* is the control surface. From a dimensional analysis, we can define the effective sedimentation rate at which the final deposit builds up (*S_p_*) as

Inverting the relationship of the deposit temperature and using the last one, we relate the sedimentation rate and the deposit temperature through the following

$$S_{p}=\frac{T_{dep}}{T_{p}}\frac{h\rho_{p}}{t}$$

*T_dep_*/*T_p_* ratio represents the rate of cooling of the deposit only due to sedimentation, however other factors can affect the final particle temperature.

| **Ignimbrite** | **Cerro Galan (CGI)^[9, 26, 30]^** | **Pozzolane Rosse (RED)^[24,25]^** | **Peperino Albano (PNO) ^[27, 28, 29]^** |
| --- | --- | --- | --- |
| **Age (Ma)** | 2.1 | 0.460 | 0.002 |
| **Chemistry** | rhyodacite | tephrite | K-foidite |
| **Areal distribution** | radial | radial | directional |
| **Extracaldera bulk (preserved) volume (km^3^)** | 364.7 (97.2) | 59 (35) | < 1 |
| **Dispersal (preserved) area (km^2^)** | 7500 (2160) | 2826 (1600) | 75 |
| **Average thickness H (m)** | 45 | 21 | 4 |
| **Max preserved distance (km)** | 74 | 33 | 8.5 |
| **Average radius (km)** | 50 | 30 | 4.5 |
| **Circle diameter L (km)** | 100 | 60 | 9 |
| **Aspect ratio H/L** | 4.5 x 10^-4^ | 3.5 x 10^-4^ | 5 x 10^-4^ |
| **Basal fallout** | no | Sub-plinian scoria | Sub-plinian pumice |
| **Max. height climbed (at distance)** | ___ | 400 m (20 km) |  |
| **Deposit T°C** | <780->620 | >580 | 240-350 |
| **%ash < 1/16 mm** | > 20 % | < 10% | > 30% |

Table S1 – Comparison of characteristics of selected ignimbrites: References: ^9^Cas et al., 2011, ^26^Lesti et al., 2011; ^30^Folkes et al., 2011; ^24^Giordano et al., 2010; ^25^Giordano and Dobran, 1994; ^27^Giordano et al., 2002; ^28^Porreca et al., 2003; ^29^Porreca et al., 2008.

*Calculation of the topological aspect ratio*

*
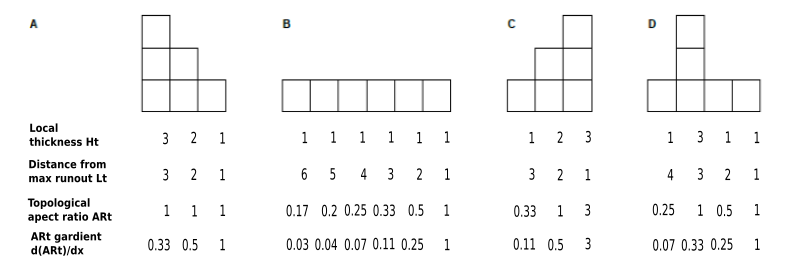
*

Fig. S1 - Method for calculation of topological aspect ratio. Proximal to distal direction is left to right. L_t_ is the distance of the site from maximum runout or from the farthest outcrop. AR_t_ gradient is calculated as the ratio between AR_t_ and L_t_.


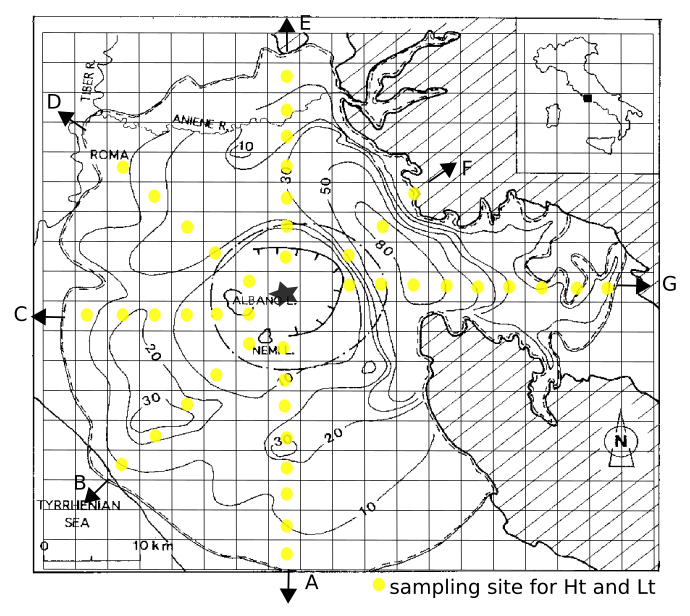
Fig. S2 – Method for sampling thickness (H_t_ ) and potential distance (L_t_) by superimposing a grid over an isopach map (modified from Giordano and Dobran 1994^24^). Arrows and letters refer to selected paths for calculation of the topological aspect ratio Ar_t_. Data in the following Table S1 (drawing realized with Inkskape ver 0.91 https://inkscape.org)


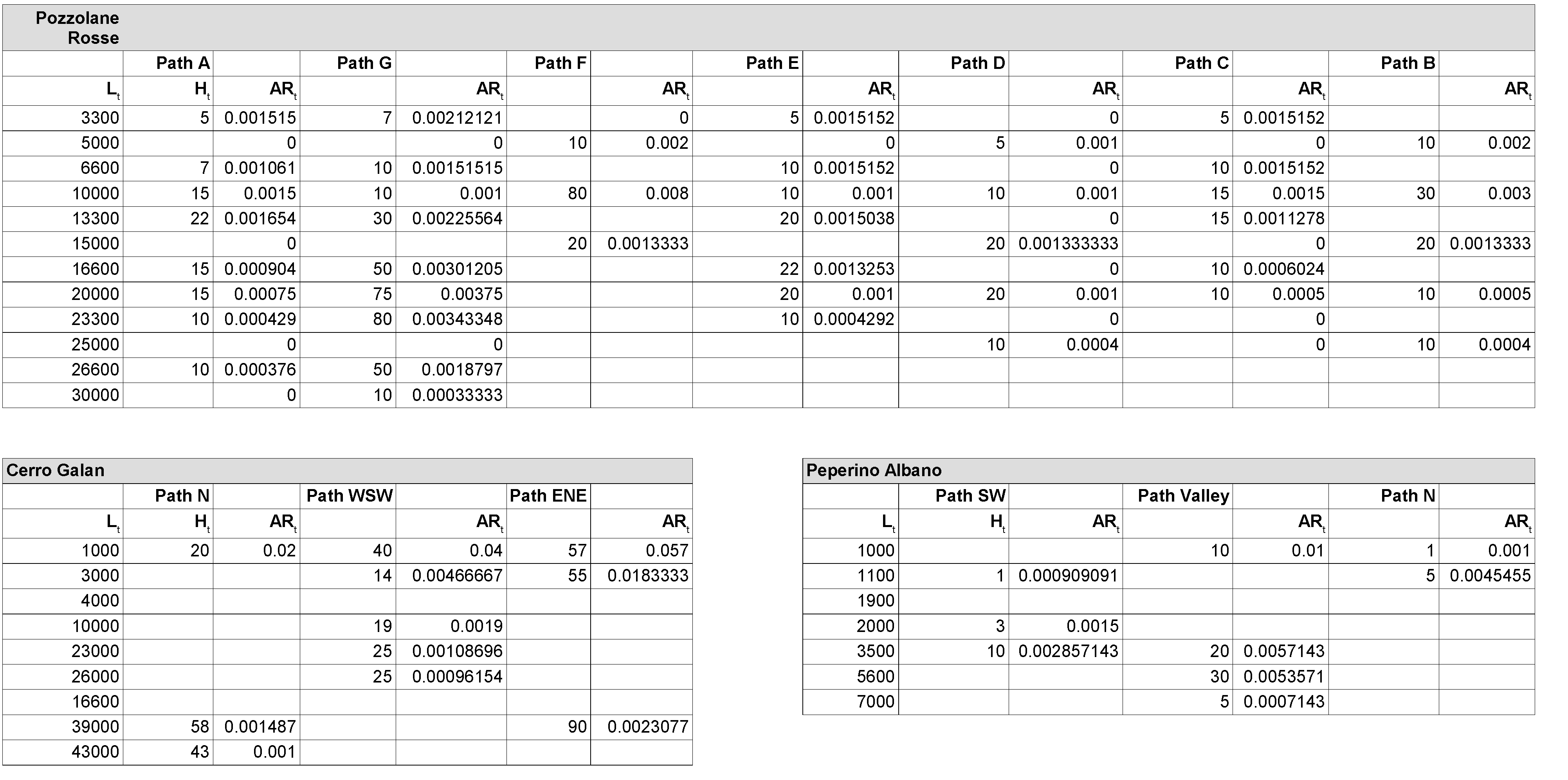


Table S2 – Data and calculations used for Figs 1b, 2b, 3b for Pozzolane Rosse, Cerro Galan and Peperino Albano ignimbrites respectively. (Paths as indicated in the figures in the main text).

*Main lithofacies of selected ignimbrites*


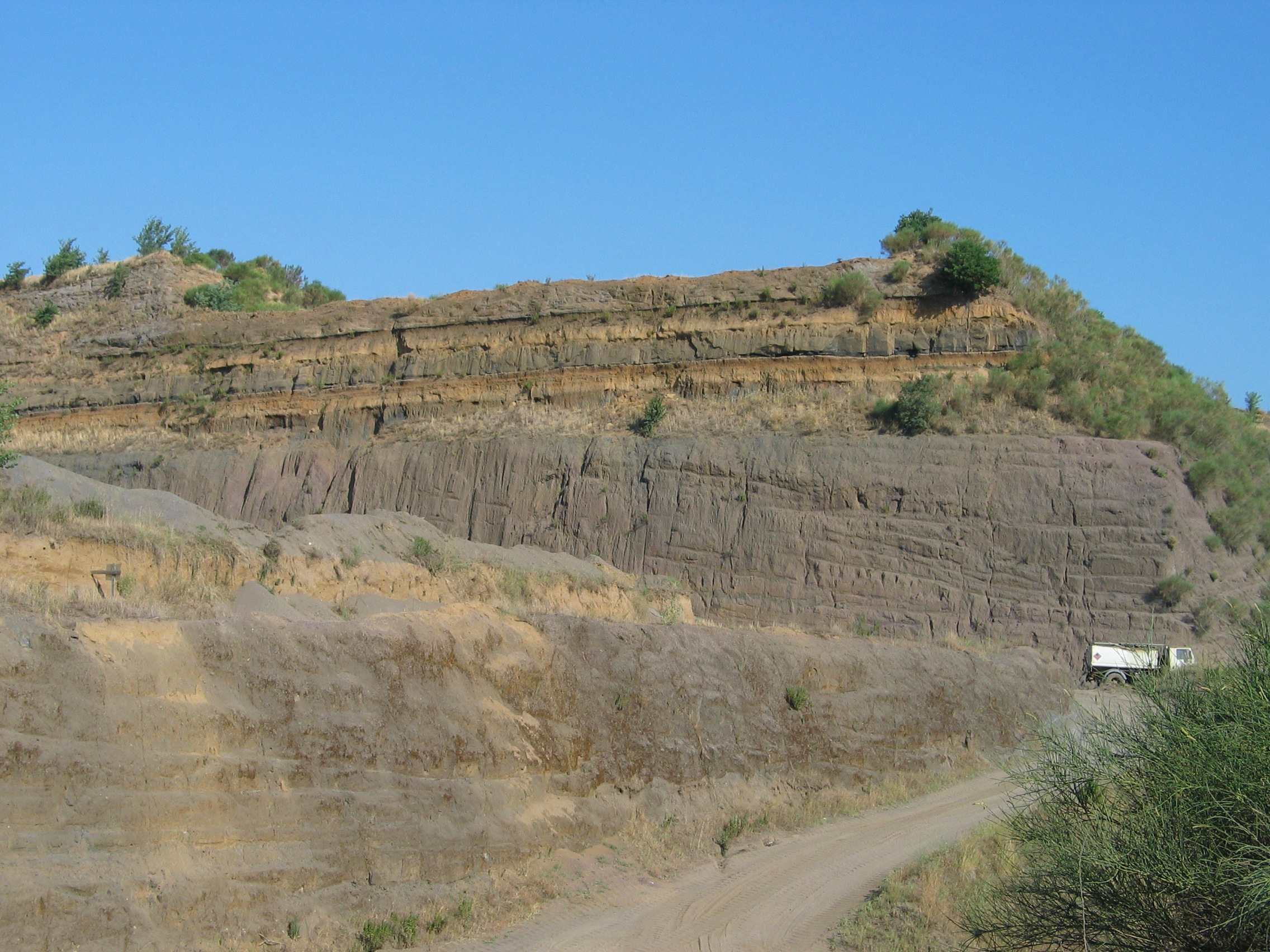

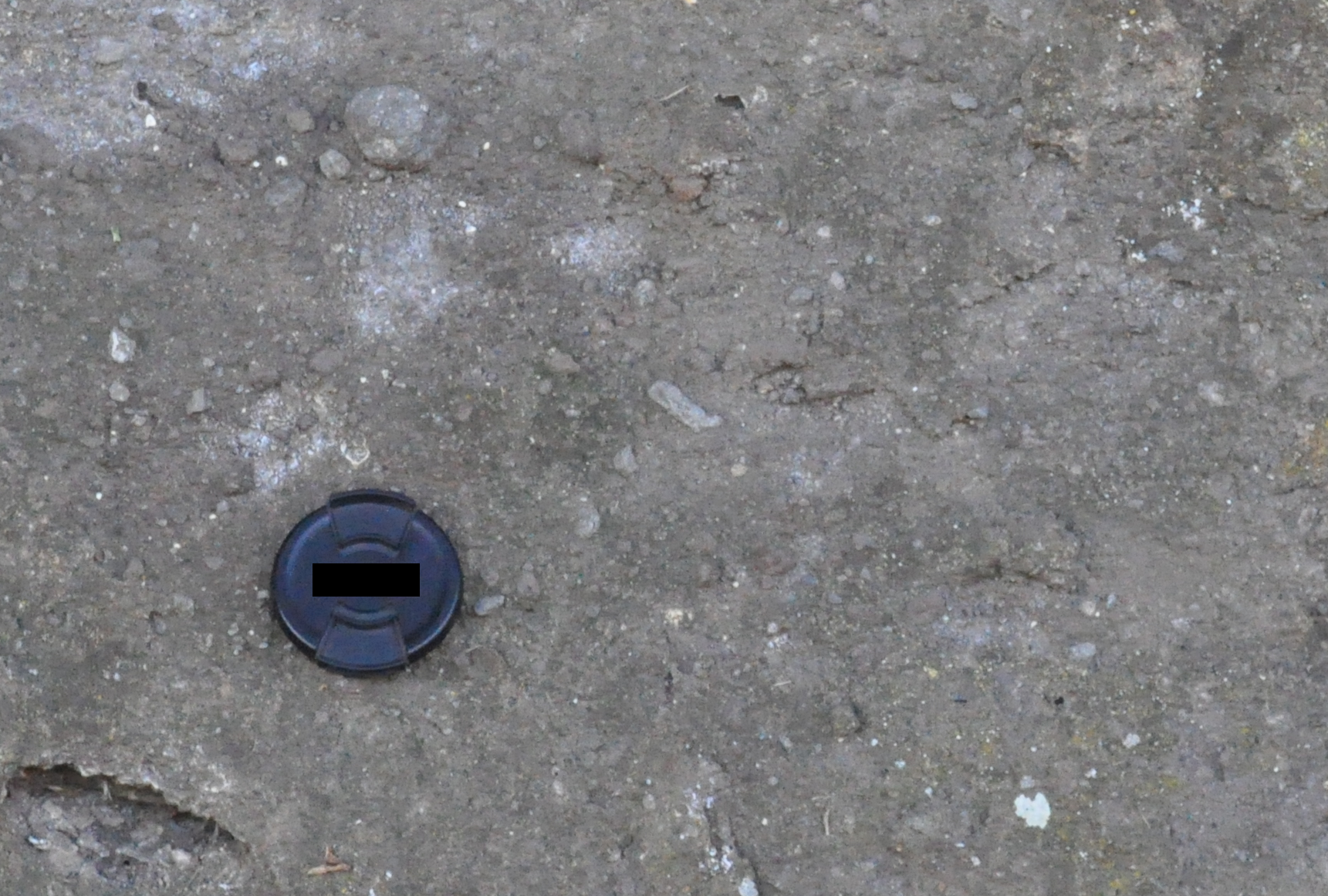


RED

**a**

**b**

**b**

Fig. S3 – a) The Pozzolane Rosse ignimbrite (RED) is, with few exceptions, a single massive and chaotic depositional unit. In this picture is more than 20 m thick (Lat. 41°55'46.05"N; Long 12°46'1.85"E); b) main massive and chaotic lithofacies (cap lens 50mm for scale).


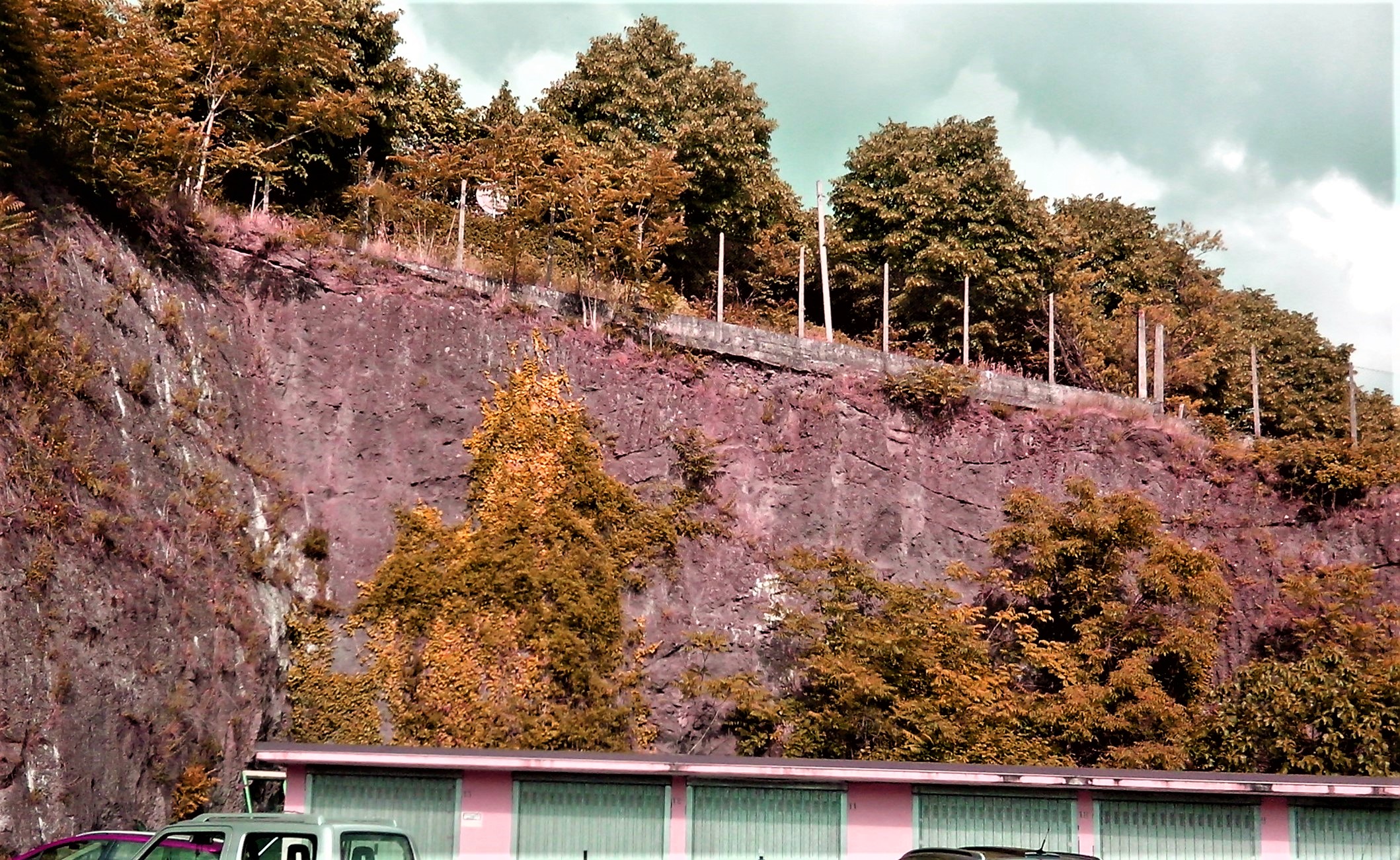

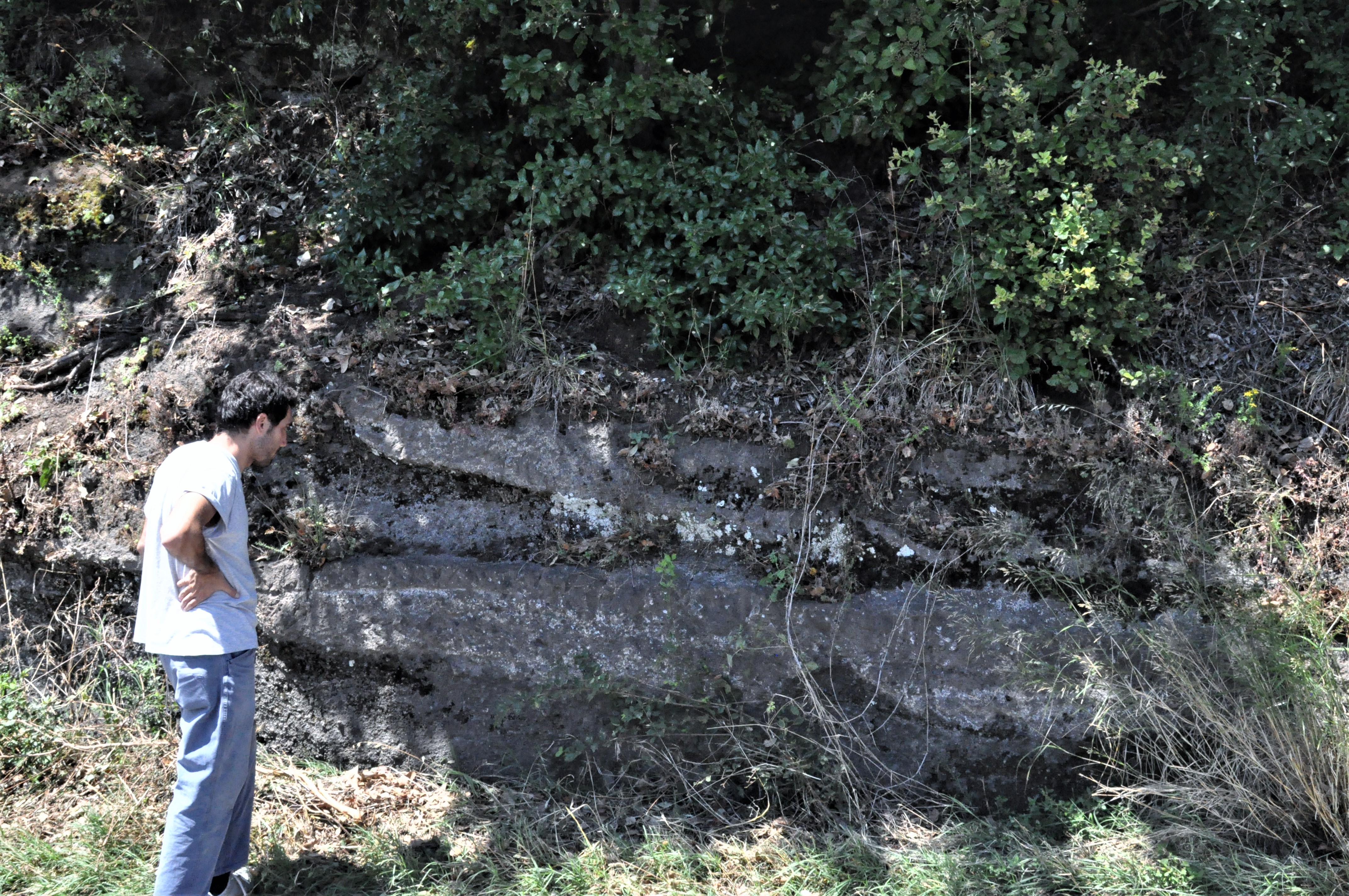
Fig. S4 – Wavy stratification and cross stratification in the Pozzolane Rosse ignimbrite are only developed beyond a major cross-sectional paleovalley to the east of the volcano, that acted as sedimentary trap; a) locality Colleferro (Lat. 41°44'18.65"N; Long. 13° 0'18.26"E); b) Locality via di Poli (Lat. 41°54'7.39"N; Long. 12°51'8.51"E)

**b**

**a**

**b**

Fig. S5 – Spectacular flat top of the Cerro Galan Ignimbrite (CGI) that pinches out against the pre-existing dome in the background (Lat. 25°54'0.98"S; Long. 67°17'47.22"W). CGI appears always as a single depositional unit.


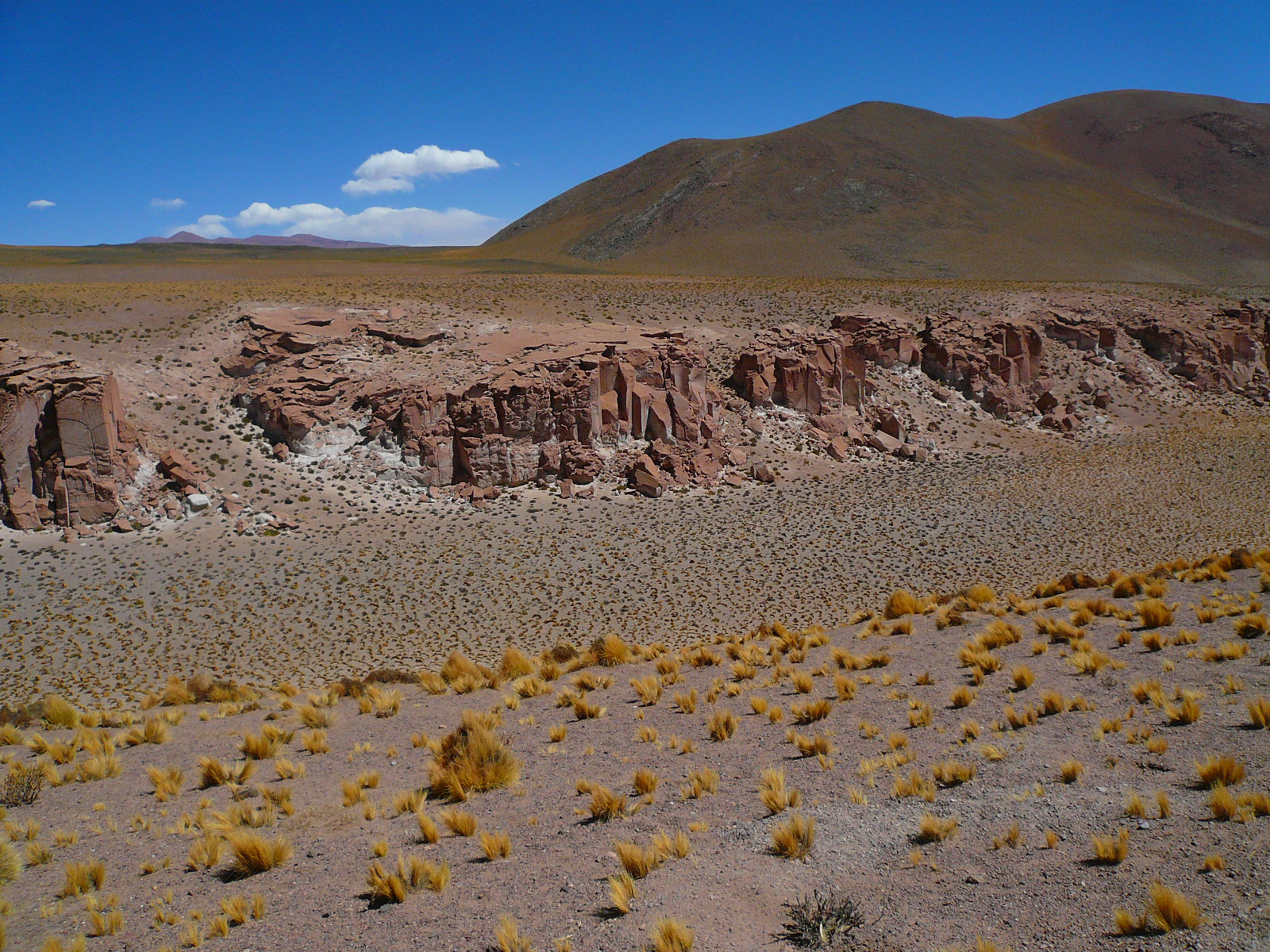

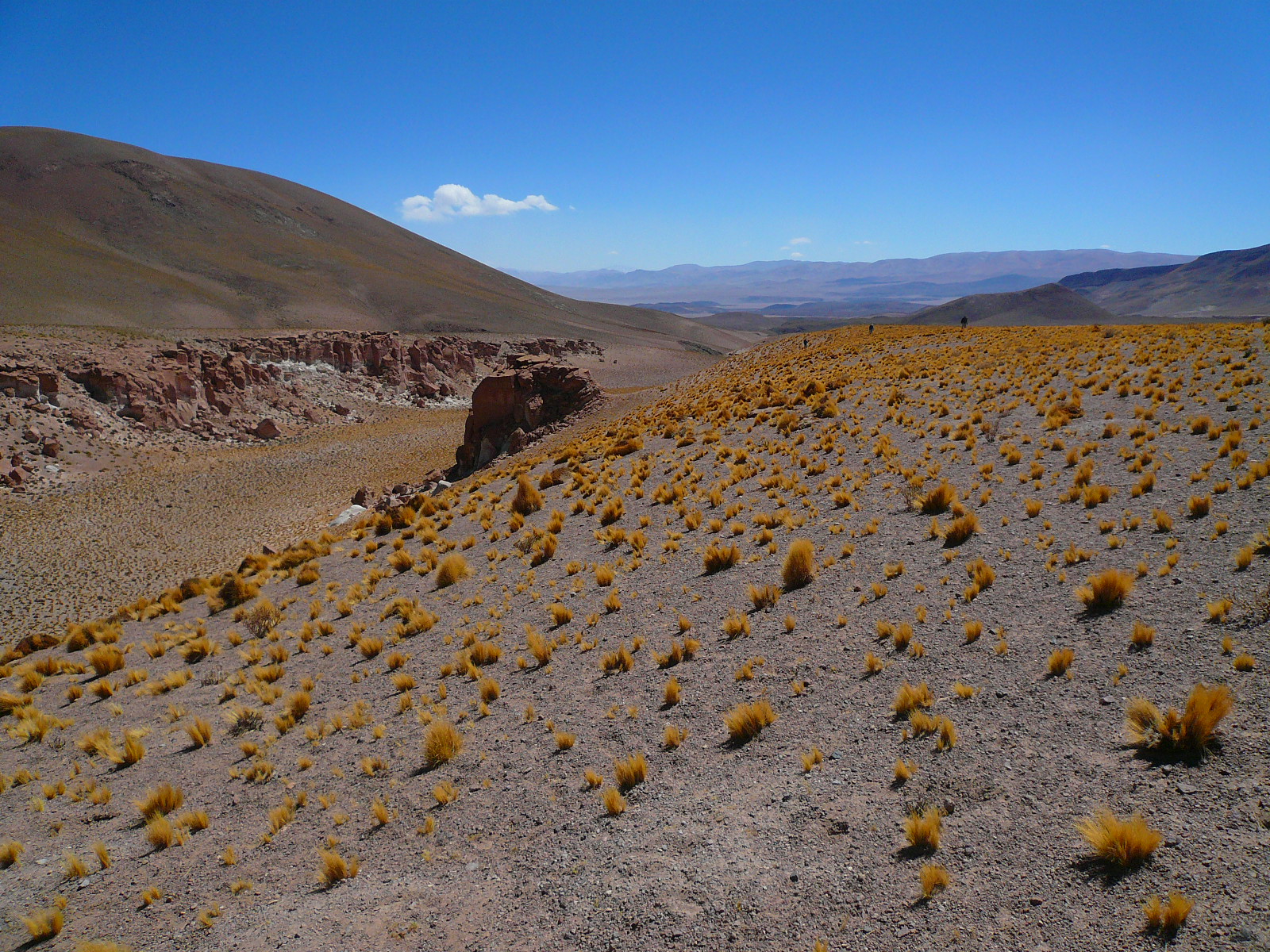


**CGI**


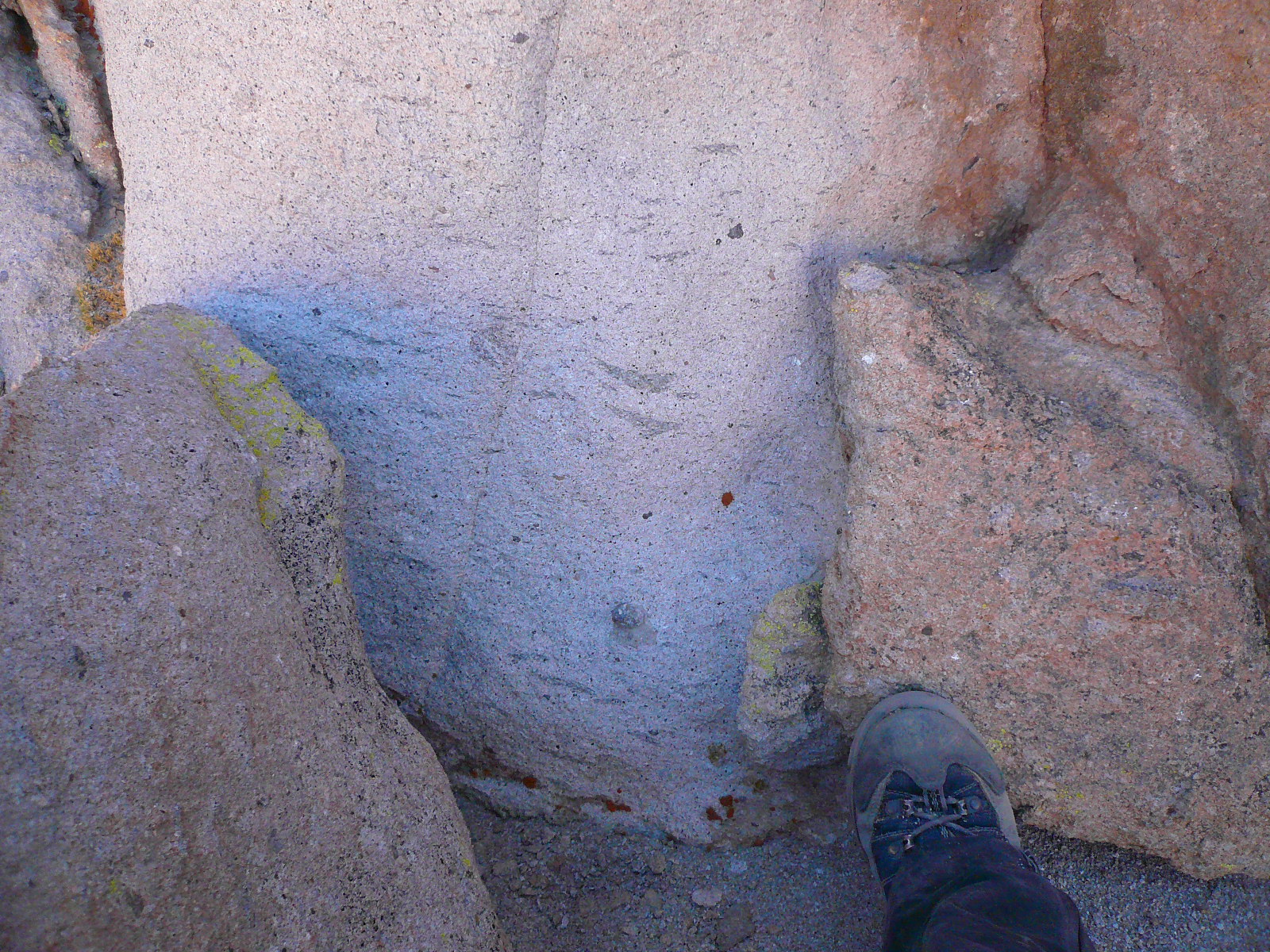
Fig. S6 – The main lithofacies of CGI is massive and chaotic, crystal rich and lithic poor, with evidence of incipient welding in proximal areas and of an internal shear fabric (clast imbrication).


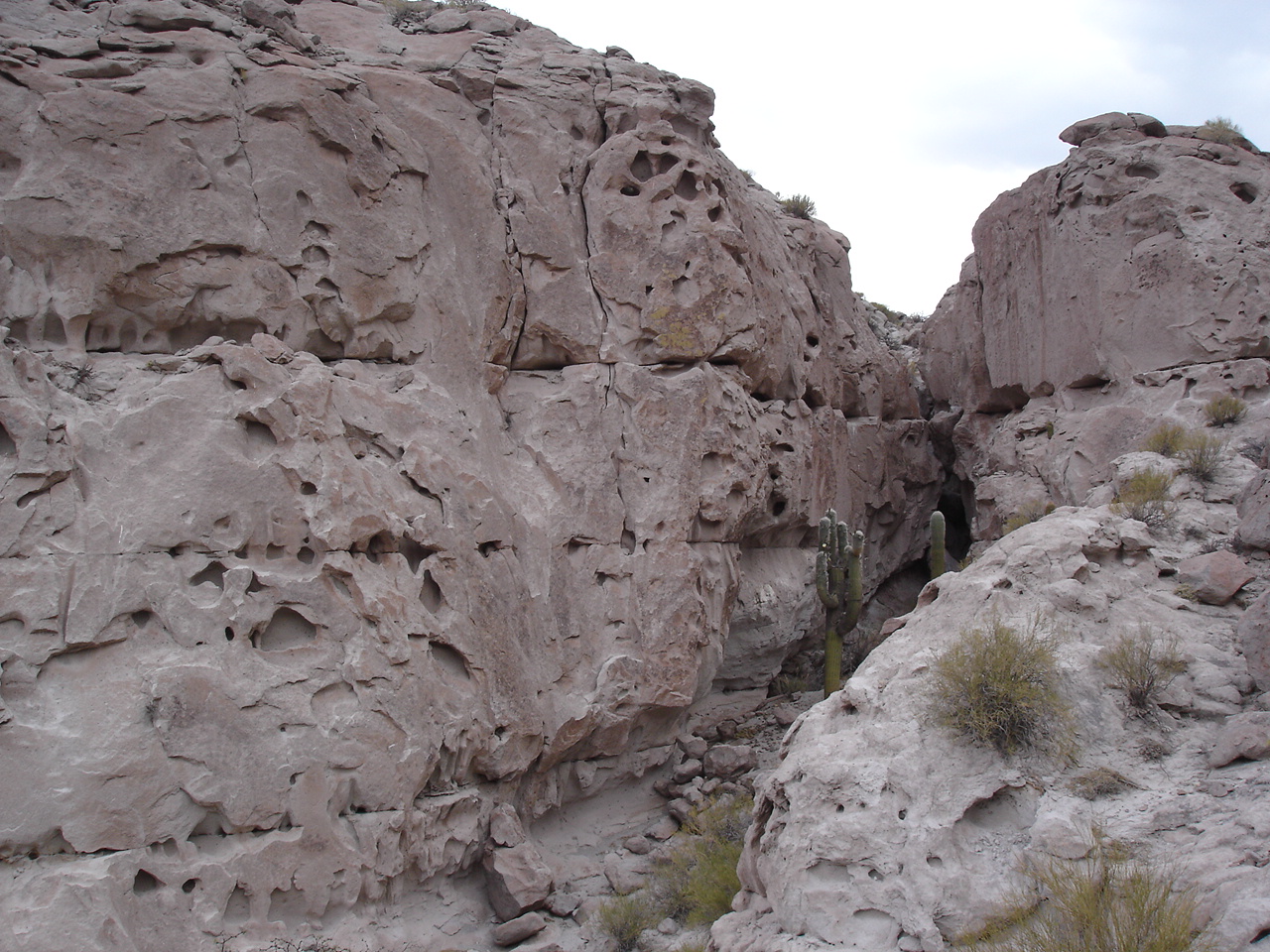
Fig. S7 – At valley ponded, distal oucrops, massive and chaotic depositional units are piled up with no signs of erosion i between (Lat. 25°41'39.12"S; Long. 66°26'4.90"W) (photo courtesy of C. Lesti)


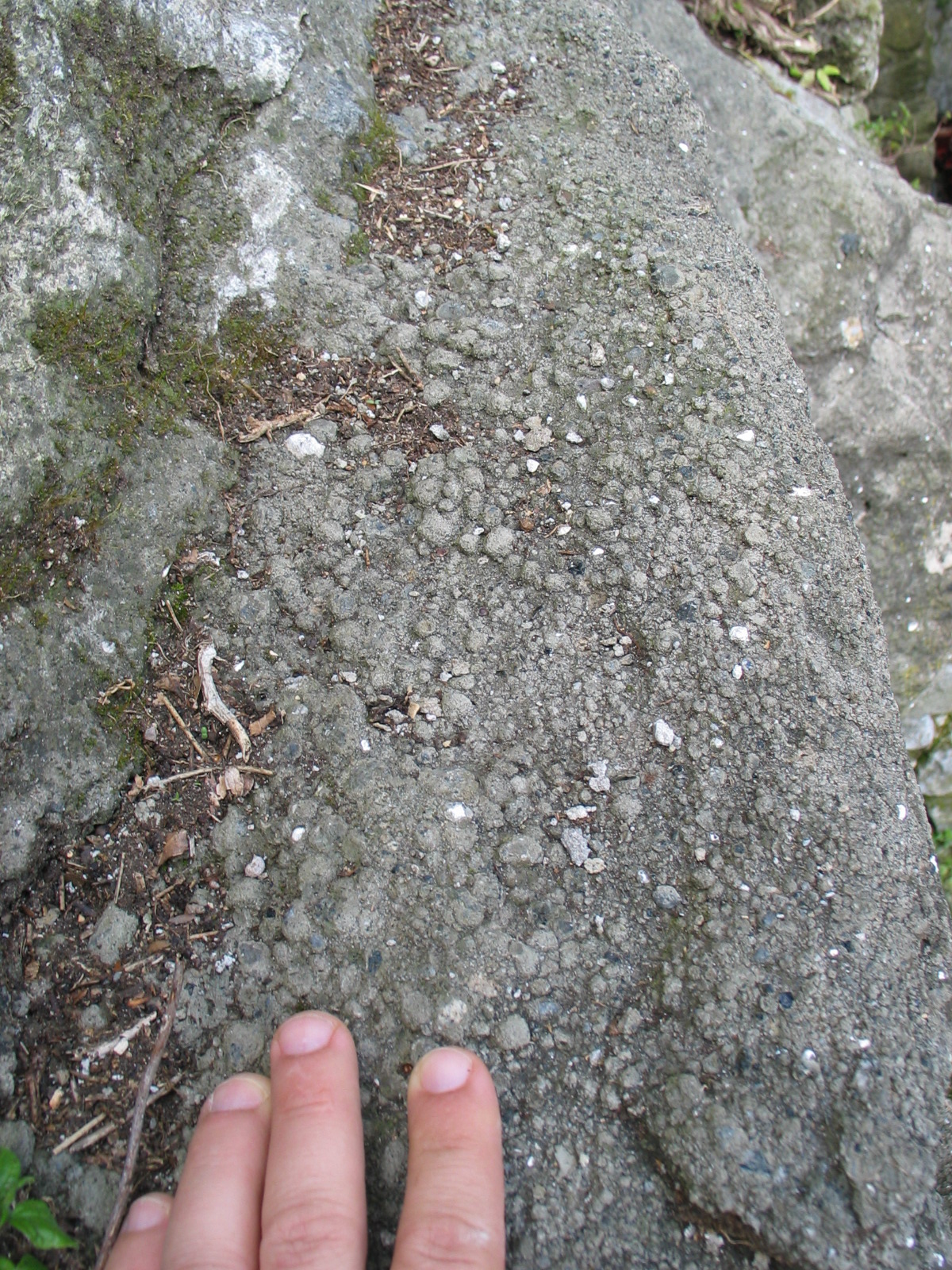

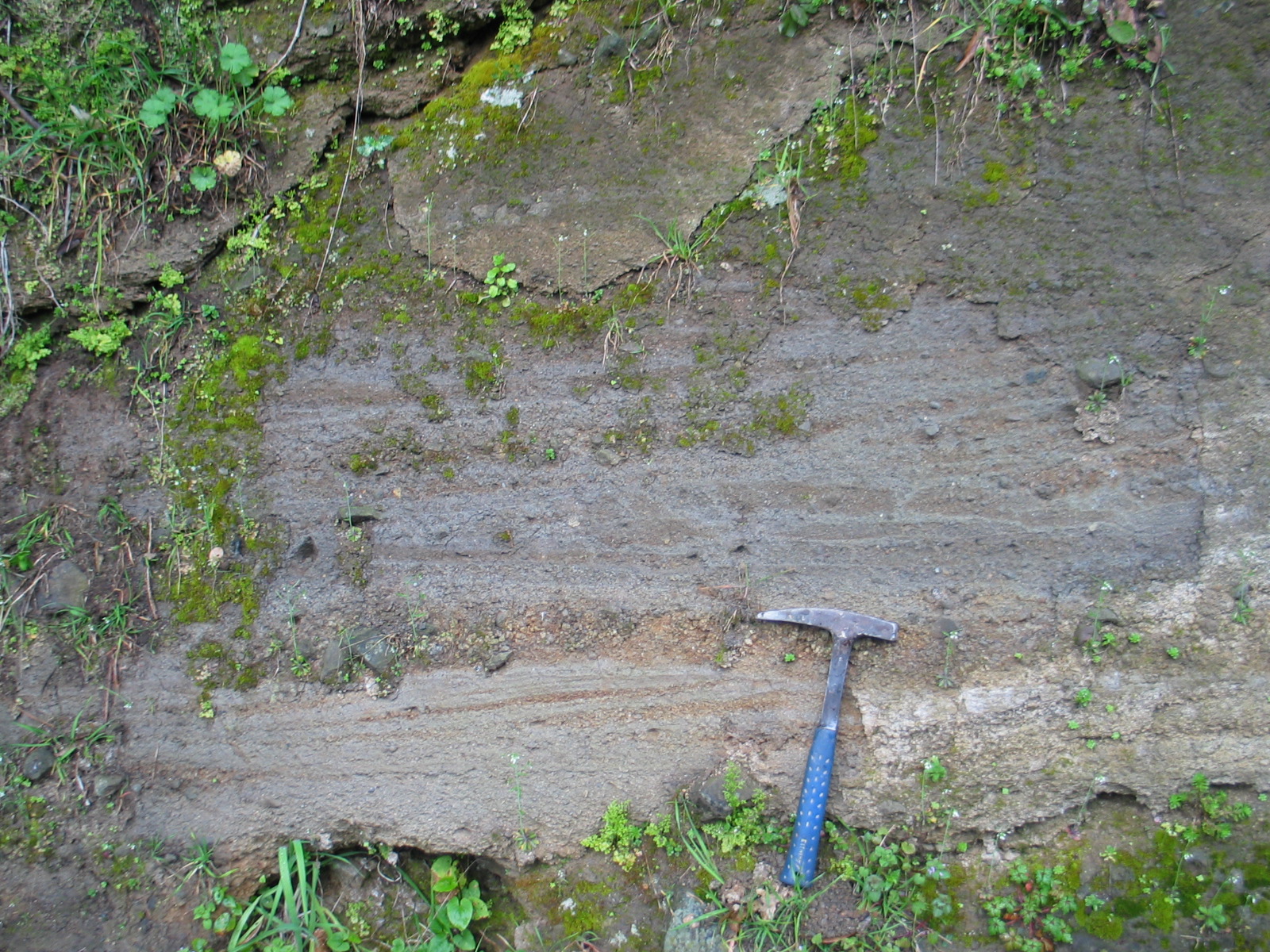
Fig. S8 – Veneer facies of the Peperino Albano ignimbrite (Lat. 41°46'9.80"N; Long. 12°39'10.47"E); a) with stratifications and b) with abundant accretionary lapilli, developed beyond the main cross-sectional valley where most of the pyroclastic debris drained to form a detached undercurrent. Accretionary lapilli are evidence of a significant temperature drop of the current that in valley confined

**b**

**a**

**b**


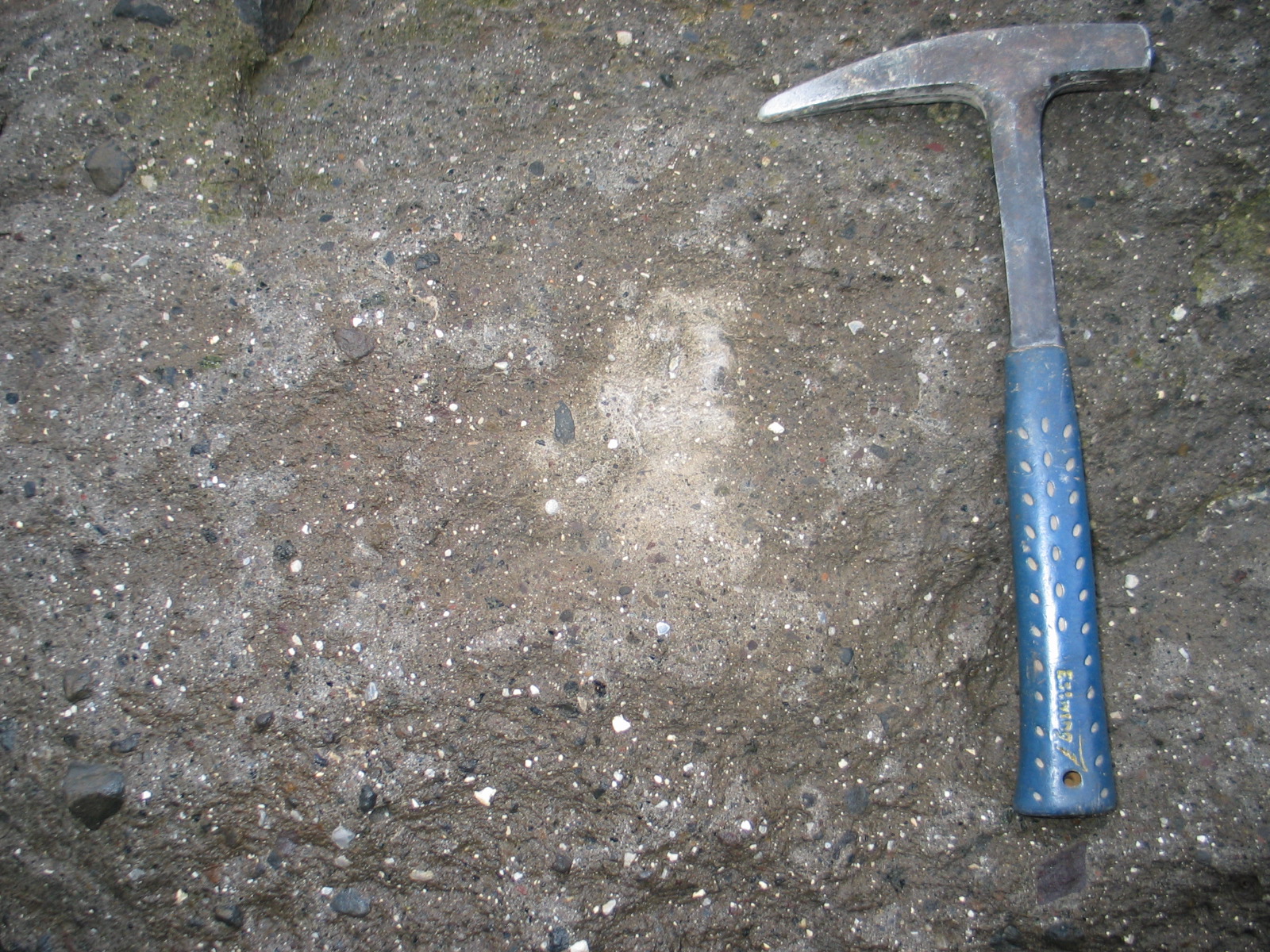


**a**


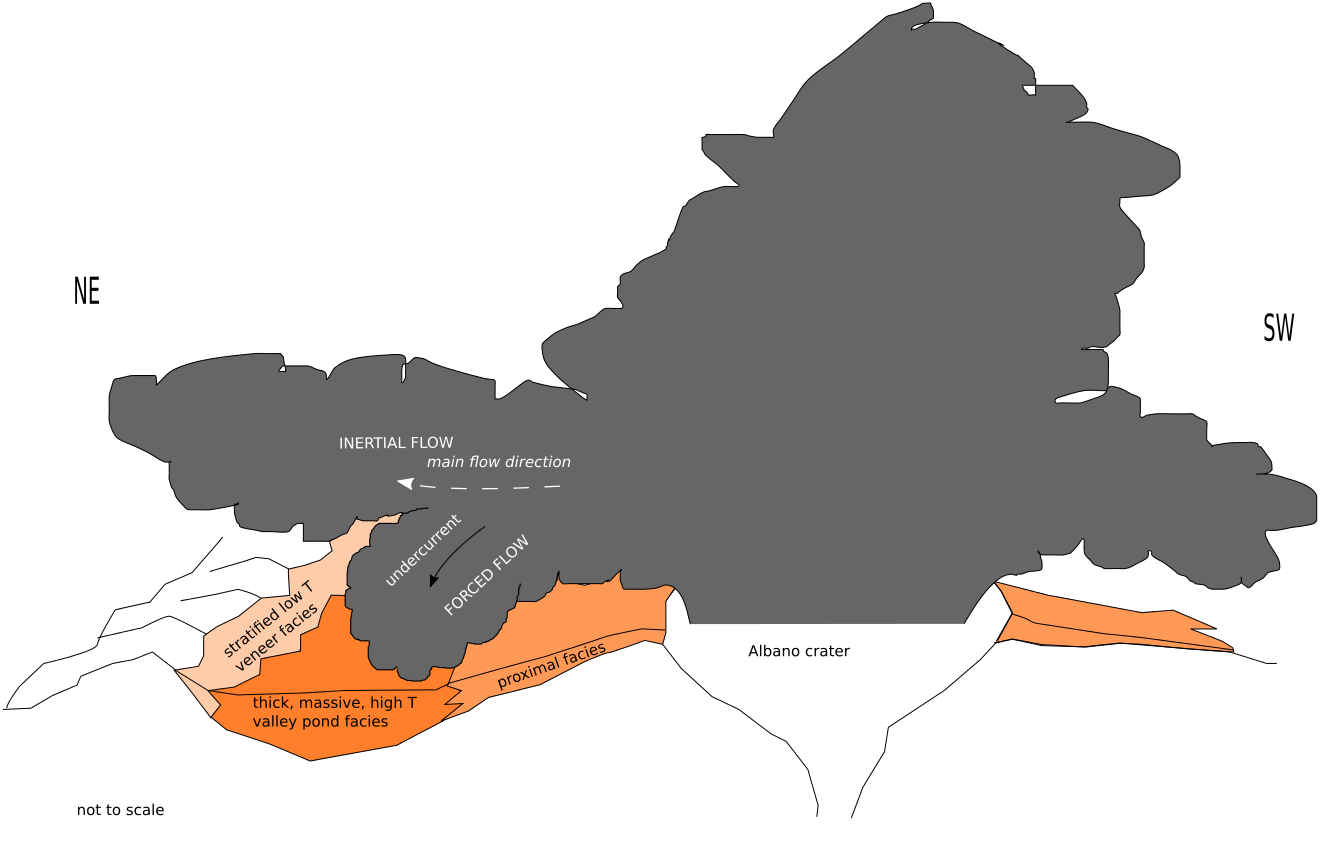
Fig. S9 – a) Proximal massive and chaotic facies of the valley pond Peperino Albano ignimbrite; b) schematic representation of the paleotopographic control on the Peperino Albano, with forced conditions within the paleovalley and inertial conditions in the veneer region across the main valley after the detachment of the high concentration undercurrent. Flow system is intentionally represented displaced from the deposit for graphic reasons.

**b**

References

[9] Cas, R.A.F., et al. The flow dynamics of an extremely large volume pyroclastic flow, the 2.08-Ma Cerro Galán Ignimbrite, NW Argentina, and comparison with other flow types: *Bull. Volcanol.* **73**, 1583-1609 (2011).

[14] Sulpizio, R., Dellino, P., Doronzo, D.M. & Sarocchi, D. Pyroclastic density currents: state of the art and perspectives: *J. Volcanol. Geotherm. Res.* **283**, 36-65 (2014).

[16] Doronzo, D.M. Two new end members of pyroclastic density currents: Forced convection-dominated and inertia-dominated. *J. Volcanol. Geotherm. Res.* **219-220**, 87-91 (2012).

[17] Roche, O., Phillips, J.C. & Kelfoun, K. Pyroclastic density currents, *in* Fagents, S.A., Gregg, T.K.P. & Lopes, R.M.C., eds., Modeling Volcanic Processes: The Physics and Mathematics of Volcanism: Cambridge University Press, 203-229 (2013).

[19] Doronzo, D.M., Martí, J., Dellino, P., Giordano, G. & Sulpizio, R. Dust storms, volcanic ash hurricanes, and turbidity currents: physical similarities and differences with emphasis on flow temperature. *Arab. J. Geosci.* **9**, 290 (2016).

[24] Giordano, G. & Dobran, F. Computer simulations of the Tuscolano Artemisio’s second pyroclastic flow unit (Alban Hills, Latium, Italy). *J. Volcanol. Geotherm. Res.* **61**, 69-94 (1994).

[25] Giordano G. & the CARG team . Stratigraphy and volcano-tectonic structures of the Colli Albani volcanic field. In: (Eds): Funiciello R. & Giordano G., The Colli Albani Volcano. vol. Geol. Soc. London, *Special Publication of IAVCEI* **3**, 43-97 (2010).

[26] Lesti, C. et al. High-temperature emplacement of the Cerro Galán and Toconquis Group ignimbrites (Puna plateau, NW Argentina) determined by TRM analyses: *Bull. Volcanol.* **73**, 1535-1565 (2011).

[27] Giordano, G., De Rita, D., Cas, R.A.F. & Rodani, S. Valley pond and ignimbrite veneer deposits in small volume phreatomagmatic basic ignimbrite, Lago Albano Maar, Colli Albani volcano, Italy: influence of topography. *J. Volcanol. Geotherm. Res.* **118**, 131-144 (2002).

[28] Porreca, M., Mattei, M., Giordano, G., De Rita, D. & Funiciello, R. Magnetic fabric and implication for pyroclastic flow and lahar emplacement, Albano maar, Italy. *J. Geophys. Res. Solid Earth* **108**, B52264 (2003).

[29] Porreca, M., Mattei, M., Giordano, G., De Rita, D. & Funiciello, R., Paleomagnetic evidence for low-temperature emplacement of the phreatomagmatic Peperino Albano ignimbrite (Colli Albani volcano, Central Italy). *Bull. Volcanol.* **70**, 877-893 (2008).

Folkes, C. B. et al. A re-appraisal of the stratigraphy and volcanology of the Cerro Galán volcanic system, NW Argentina. *Bull. Volcanol.*,***73***(10), 1427-1454 (2011).
